# Training AI to Improve Distinction of Triple-Negative Invasive Breast Cancer from Cysts and Fibroadenomas on Ultrasound

**DOI:** 10.3390/diagnostics16091354

**Published:** 2026-04-30

**Authors:** Wendie A. Berg, Andriy I. Bandos, Linda H. Larsen, Samantha L. Heller, Regina J. Hooley, Richard S. Ha, Maham Siddique, Jeremy M. Berg, Yuying Cao, R. Chad McClennan, Ajit Jairaj

**Affiliations:** 1 Department of Radiology, University of Pittsburgh School of Medicine, Pittsburgh, PA 15213, USA; 2 Department of Biostatistics and Health Data Science, University of Pittsburgh School of Public Health, Pittsburgh, PA 15261, USA; 3 Department of Radiology, Keck School of Medicine of USC, Los Angeles, CA 90033, USA; lindahlarsen@dhs.lacounty.gov; 4 Department of Radiology, NYU Grossman School of Medicine, NYU Langone Health, New York, NY 10016, USA; 5 Department of Radiology and Biomedical Imaging, Yale School of Medicine, New Haven, CT 06520, USA; 6 Department of Radiology, Columbia University School of Medicine, New York, NY 10032, USA; 7 Department of Computational and Systems Biology, University of Pittsburgh School of Medicine, Pittsburgh, PA 15213, USA; 8 School of Medicine, University of Pittsburgh, Pittsburgh, PA 15213, USA; 9 Koios Medical, Inc., Chicago, IL 60654, USA

**Keywords:** breast ultrasound, artificial intelligence, triple-negative invasive breast cancer, BI-RADS, complicated cysts

## Abstract

**Background/Objectives:** Circumscribed oval, hypoechoic masses are common on screening breast ultrasound (US), and the vast majority are benign. Triple-receptor negative invasive breast cancer (TNBC) can appear similar, resulting in both human and artificial intelligence (AI) interpretive errors. **Purpose**: We sought to improve AI performance in distinguishing common benign masses from TNBC through a retrospective model refinement and validation study. **Materials and Methods**: In an Institutional Review Board-approved HIPAA-compliant protocol, from five academic medical centers, orthogonal ultrasound images of 1771 breast masses 2 cm or smaller were acquired, consisting of cysts, complicated cysts, other benign, and malignancies. Cases were randomized, controlling for lesion class, site, and patient age, with 1446 (including 402, 27.8%, malignancies) used for training and 325 (including 95, 29.2% malignancies) for validation using Koios DS^®^ (decision support, KDS) software version 2.0. A breast imaging radiologist from each center reviewed images and recorded BI-RADS features and assessment. Demographics, symptoms, and pathology or at least one-year follow-up was recorded. The KDS score was evaluated standalone and in combination with BI-RADS using logistic regression and ROC analysis with focus on specificity at sensitivity of 98%. **Results**: In training, KDS standalone performed comparably to BI-RADS, and significantly improved BI-RADS malignancy risk prediction (*p* < 0.001). The 98%–sensitivity threshold for combined KDS + BI-RADS was estimated and kept fixed during validation. In validation, KDS standalone performed similar to BI-RADS with AUC = 0.97 (CI: 0.95–0.98) versus 0.95 (*p* = 0.22), with sensitivity of 98% (93/95, CI: 95–100%) for both and specificity of 70.9% (163/230, CI: 65.0–76.7%) for KDS versus 63.9% for BI-RADS (147/230, *p* = 0.10). Combining KDS + BIRADS significantly improved overall performance (AUC 0.98, *p* < 0.001) and specificity (74.4%, 171/230, *p* < 0.001) while maintaining sensitivity at 98% (93/95). **Conclusions**: While KDS alone should not replace BI-RADS, when used in combination with BI-RADS, it can significantly improve specificity for highly accurate (98% sensitivity) triaging management of masses representative of those seen on screening US.

## 1. Introduction

With increasing awareness of the masking of cancers in dense breasts and breast density notification, including a national standard in the United States effective since 2024, there is growing interest in supplemental screening beyond mammography [1,2,3,4,5,6,7,8,9,10,11]. While MRI is the most sensitive test for breast cancer [3,10,12,13], screening MRI is often not well tolerated [14,15,16] or accessible. Contrast-enhanced mammography (CEM) may prove to be a good alternative to MRI [1,7,17], but is not yet approved by the Food and Drug Administration for screening. Ultrasound (US) has been widely validated for supplemental screening of women with dense breasts [2,18], and does not require contrast injection or ionizing radiation. US performance as a screening test is hampered, however, by the high prevalence of probably benign and low-suspicion masses, often requiring short-interval follow-up or biopsy.

BI-RADS 3, probably benign masses, have less than 2% risk of malignancy and include a solitary oval circumscribed hypoechoic or isoechoic mass on baseline screening, as on mammography, and also new isolated solid circumscribed oval masses seen on annual screening US [19]. Clustered microcysts have at times been included as BI-RADS 3 findings but can usually be dismissed as benign, BI-RADS 2 [20]. Of 2662 participants in the American College of Radiology Imaging Network (ACRIN) 6666 study, 519 (19.5%) had 745 BI-RADS 3 masses on screening US (representing 25.5% of the 2916 lesions other than simple cysts) [21]. Of the 745 BI-RADS 3 masses, 124 (16.6%) ultimately underwent biopsy and six (0.8%) proved malignant. Another 400 low-suspicion BI-RADS 4A lesions were identified on screening US, of which 330 (82.5%) underwent biopsy and 11 (2.8%) proved malignant. Across the literature, 8/3918 (0.2%) BI-RADS 3 masses on screening US were malignant at 6-month follow-up, as were 17/4364 (0.39%) at 2-year follow-up [19]. With such a low malignancy rate, comparable to BI-RADS 2 findings, annual follow-up of BI-RADS 3 findings at the time of annual screening US has been suggested as a safe alternative to short-interval follow-up [19]. The concern is that some high-grade malignancies, particularly triple-receptor negative invasive breast cancer (TNBC), can appear circumscribed and oval on US [22] and be misinterpreted as probably benign or even benign. Even a 6-month delay in diagnosis of such tumors could adversely affect prognosis.

AI applied to breast US can reduce false positive biopsies, improve sensitivity, and potentially improve accuracy [23]. Koios DS^®^ (decision support, KDS) AI software is used to assist radiologists in assessing masses seen on breast US and has been shown to improve diagnostic performance [24]. When applied to masses from screening US, prior work showed KDS performance was comparable to specialists in breast imaging, with the same cancers assessed as probably benign by radiologists also assessed as probably benign by KDS software [25]. In this work, we sought to improve performance of KDS distinguishing TNBC from the common benign and low-suspicion sonographic masses representative of those seen on screening US. In particular, we expected that re-trained KDS would offer improvements in AUC, as well as in specificity, at a sensitivity of 98% (making it suitable for triaging tasks).

## 2. Methods

In an Institutional Review Board-approved Health Insurance Portability and Accountability Act-compliant protocol, with waiver of informed consent, orthogonal deidentified US images were retrospectively identified for 1786 breast masses (one per patient) from five academic medical centers in the USA. Sites were instructed to collect images and clinical information on four classes of lesions: cysts, complicated cysts with debris, other benign masses (mostly fibroadenomas), and TNBC; 23 relatively circumscribed estrogen receptor (ER)- and progesterone receptor (PR)-positive, or human epidermal growth factor-2 (HER2)-positive malignancies were also included in an effort to improve and test AI performance for low-suspicion masses. Ultrasound equipment from at least 7 manufacturers was represented, and linear array transducers with a center frequency of at least 12 MHz were used for all images. All masses had biopsy (including excision after an atypical result on core biopsy) or at least one year of follow-up with stability/no cancer diagnosis; BI-RADS 3 masses not biopsied either decreased or resolved or had at least two years of imaging stability. Benign masses were selected from screening US or mammography cases, and all masses were intended to be representative of those seen on screening US, with maximum diameter of 20 mm: 14 masses were excluded due to size exceeding 20 mm and one due to erroneous upload of normal images of a different area of the breast, leaving 1771 for analysis.

Cases were randomized, controlling for lesion class, site, and patient age, with 1446 (81.6% of cases) used for training and 325 (18.4%) held for validation testing of the AI, Koios DS^®^ Decision Support (KDS) v. 2.0, Koios Medical, Inc., New York, NY, USA, a commercially available, FDA-approved, CE-marked software as a medical device. KDS employs a deep neural network trained using a supervised learning approach. The model had been trained on a large dataset of over one million breast ultrasound images with pathologically confirmed diagnoses serving as ground truth labels, and high reliability of outputs has been shown [24]. The size of the validation set was planned to provide statistical power for detecting the expected differences in the AUCs as well as large differences in specificity at 98% sensitivity. Authors who are not employees of Koios Medical, Inc. retained full control of the data, analysis, and presentation of results.

Five radiologist specialists, each with at least 10 years’ experience in breast US, reviewed all images and recorded BI-RADS feature descriptors through online software developed for this study. The original clinically reported BI-RADS diagnostic final assessments were used unless they were ambiguous due to multiple modalities or findings; in such rare cases with ambiguity, the reviewer’s final assessment was then recorded for the lesion based on US features. We collected patient age, race, BI-RADS visual mammographic breast density, how the mass was found (screening mammogram, palpable, screening US, MRI finding), US largest diameter, and results of core biopsy and surgery (if available).

Raw quantitative KDS outputs were scaled monotonically to ensure that the same output space was used for each of four quartiles from 0 to 1.0. The scaled scores were used for analysis, with scores of <0.25 and ≥0.25 to <0.50 scaled to “benign” and “probably benign” outputs respectively and considered negative; scores of ≥0.50 to <0.75 and ≥0.75 to 1.0 were scaled to “suspicious” and “probably malignant” assessments respectively and were considered positive. Diagnostic BI-RADS assessments of BI-RADS 4A or higher were considered positive. The primary endpoint was the imputed performance of BI-RADS plus the quantitative KDS score in the validation set.

### Statistical Analysis

The primary analysis focused on evaluating KDS performance in classifying lesions as benign or malignant, and more specifically, on ruling out malignancy, in standalone mode, compared to BI-RADS alone. We then evaluated performance of the derived addition to BI-RADS via the combination of the KDS score with clinical BI-RADS assessments (KDS + BI-RADS). The primary outcomes were area under the ROC curve (AUC), and specificity at sensitivity of at least 98%. The ability of KDS to accurately classify benign and malignant lesions among BI-RADS 2, 3, and 4A lesions after training was also of interest.

The added value of KDS for benign/malignant classification beyond the clinical BI-RADS assessment alone was statistically tested using multivariable logistic regression. Logistic regression was also used to estimate a simple and robust, proof-of-concept, combination of KDS + BI-RADS (without aiming for the optimal combined marker). The combined KDS + BI-RADS assessment and its threshold for achieving 98% sensitivity were derived in the training set and applied unchanged in the validation set. The primary statistical evaluation was performed on the validation set, with performance in the training set evaluated for consistency. We examined the impact of symptoms present, lesion size, and age on AUC to assess the consistency of the overall effects. Two-sided 95% confidence intervals (CI) and *p*-values for paired data statistical tests were determined for relevant assessments using DeLong’s test for AUCs and McNemar’s test for proportions. All analyses were performed using SAS statistical software (version 9.4, SAS Institute, Cary, NC, USA).

## 3. Results

The total sample comprised 1771 masses in 1771 women, median age 54 years (range 22–97). Of the 1771 masses, 497 (28.1%) were malignant with median size 12 mm (range 2 to 20 mm, IQR: 5 to 12 mm) and 433/1771 (24.4%) were symptomatic (including 203/497, 40.8% malignancies). Among the 497 malignancies, 475 (95.6%) were TNBC [449 invasive ductal carcinomas (IDC), 12 invasive lobular carcinomas (ILC), eight mixed IDC-ILC, five invasive special types (two adenoid cystic, and one each metaplastic, invasive papillary, and mucinous), and one metastatic intramammary lymph node]. Eleven malignancies were IDC ER/PR+, HER2−; eight were ductal carcinoma in situ (DCIS); one was ILC ER/PR+, HER2−; one was ILC ER/PR-, HER2+; and two were encapsulated papillary carcinoma (EPC). All malignancies and 520/1274 (40.8%) benign lesions underwent biopsy or aspiration. Of the 1274 benign masses, there were: 530 complicated cysts with debris; 470 simple cysts; 104 fibroadenomas; 96 fibrocystic changes; 32 clustered microcysts; 10 ruptured cysts; 5 fat necrosis; 4 apocrine metaplasia; 4 papillomas; 2 intramammary lymph nodes; 2 epidermal inclusion cysts; 1 each of abscess, fibrosis, pseudoangiomatous stromal hyperplasia, phyllodes tumor (excised), atypical ductal hyperplasia (excised); and 10 missing details.

In the overall case set, there were 565 BI-RADS 2 masses (with 2, 0.4%, malignant), 222 BI-RADS 3 masses (with 4, 1.8%, malignant) and 527 BI-RADS 4A masses (with 74, 14.0%, malignant). There were also 175 BI-RADS 4B masses (with 145, 82.9%, malignant), 119 BI-RADS 4C masses (with 109, 91.6%, malignant), and 163 BI-RADS 5 masses (all malignant). Malignant lesions had significantly higher BI-RADS assessments (*p* < 0.001), were more frequently larger than 1 cm (58%, 287/497, vs. 23%, 299/1274, *p* < 0.001) and symptomatic (41%, 203/497, vs. 18%, 230/1274, *p* < 0.001), and were more often observed in older (*p* < 0.001) and Black (18%, 91/497, vs. 8%, 97/1274, *p* < 0.001) women (Table 1).

### 3.1. How Common Were Benign Features in Triple-Negative Carcinomas?

**Figure 1:**
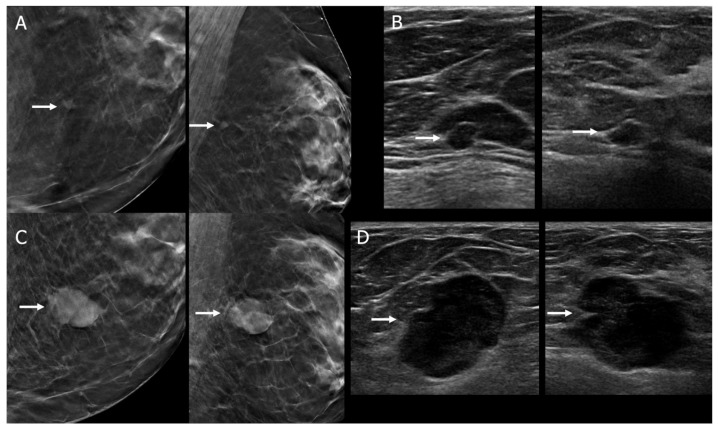
**Potential earlier diagnosis of triple-negative invasive ductal carcinoma (TNBC) by AI.** This 54-year-old woman was recalled from screening tomosynthesis for a new nodule upper inner left breast. (**A**) Close-up craniocaudal (CC) and mediolateral oblique (MLO) tomosynthesis images of the left breast show a 6-mm circumscribed mass (arrows) in the upper inner left breast, new compared to prior mammograms (not shown). (**B**) Targeted radial and antiradial US images show a corresponding circumscribed oval 5-mm anechoic mass (arrows) left breast 11 o’clock position 5 cm from the nipple, with no internal vascularity (Doppler not shown), thought to be a simple cyst, BI-RADS 2. This mass was assessed as suspicious on KDS training (score of 0.57). (**C**) Close-up CC and MLO tomosynthesis images from screening mammogram left breast, 18 months later, show moderate enlargement of the now lobulated mass (arrows), now measuring 22 mm on (**D**), radial and antiradial US images. This proved to be a 2.4-cm grade 3 TNBC that showed partial response to neoadjuvant chemotherapy. One sentinel node was negative for metastatic disease.

**Figure 2:**
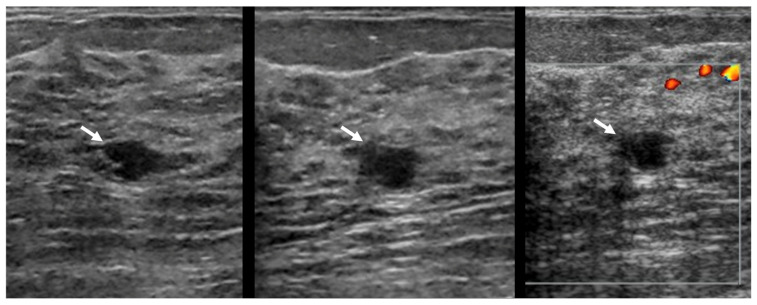
**False-negative AI assessment of TNBC.** Targeted sagittal (**left**), transverse (**middle**), and transverse power Doppler (**right**) US images directed to a suspicious enhancing mass on MRI in this 48-year-old woman show an oval, circumscribed, anechoic 6-mm mass (arrows) with posterior enhancement and no internal vascularity. This was assessed as BI-RADS 4B due to the context of a correlating enhancing mass on MRI, but, in the validation set, the KDS score was 0.27 (a probably benign rating). Core biopsy showed grade 3 TNBC.

**Figure 3:**
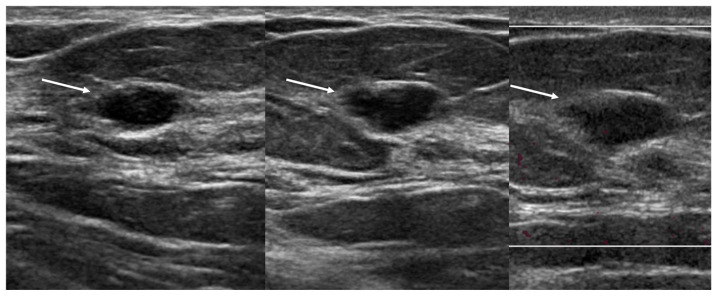
**False-negative AI assessment of TNBC.** This 48-year-old woman was recalled from screening tomosynthesis (not shown) for a new, indistinctly marginated mass left breast. Targeted transverse (**left**), sagittal (**middle**), and sagittal power Doppler (**right**) US images show an oval 7-mm anechoic mass described as a possible complicated cyst on US review, but clinically assessed as BI-RADS 4B due to mammographic features. In the validation set, the KDS score was 0.41 (a probably benign rating). Core biopsy showed grade 3 TNBC.

Among 475 TNBC 2 cm or smaller, 287 (60.4%) were described as irregular, and therefore suspicious. There were 145 TNBC masses described as having oval shape, of which margins were circumscribed in 34, indistinct in 63, microlobulated in 26, angular in 16, and spiculated in six. Eight were described morphologically as special cases, including five appearing to be complicated cysts with debris, two clustered microcysts, and one a simple cyst (Figure 1). Another 34 had round shape, with margins as follows: circumscribed in 12, indistinct in 18, microlobulated in three, and angular in one. Thus, 54/475 (11.4%) TNBC could be considered to have relatively benign features (i.e., oval or round shape and circumscribed margins or special cases). Of the 475 TNBC, 472 (99.4%) were assessed as BI-RADS 4A or higher clinically, including 53/54 (98.1%) with benign features. On KDS, 458/475 (96.4%) TNBC were correctly assessed as suspicious or malignant, including 46/54 (85%) with benign features. Of 17 TNBC misclassified as benign or probably benign by KDS, eight had benign features: six were described as oval, circumscribed masses (including one in the validation set, Figure 2); another was described as a complicated cyst (in the validation set, Figure 3) and one other as clustered microcysts.

### 3.2. Training Performance

In the training set of 1446 cases, including 402 (27.8%) malignancies, the quantitative KDS score had an AUC of 0.96 (CI: 0.95–0.98) and achieved 95.3% (383/402, CI: 93–97%, i.e., lower than 98%) sensitivity and 77.8% (812/1044) specificity at the default threshold of 0.50. Sensitivity of 98% (394/402) was achieved at a threshold of 0.23, resulting in specificity of 61.8% (645/1044). The BI-RADS assessments, despite their categorical nature, had a practically equivalent AUC of 0.96 (95%CI, 0.95–0.97), with sensitivity 99.0% (398/402) and specificity 60.7% (634/1044) for the clinically actionable findings (i.e., BI-RADS 4A or higher).

**Figure 4:**
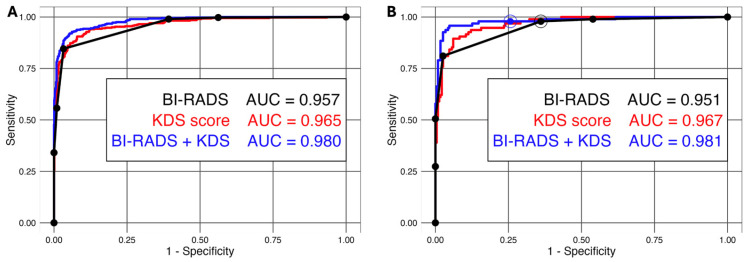
**ROC curves for assessing sonographic masses** for (**A**) training set of 1044 benign and 402 malignant lesions and (**B**) validation set of 230 benign and 95 malignant lesions. Note circles on cut-points for the thresholds of BI-RADS 4A or higher, or BI-RADS + DS at empirical threshold of 16.5 for 98% sensitivity.

**Figure 5:**
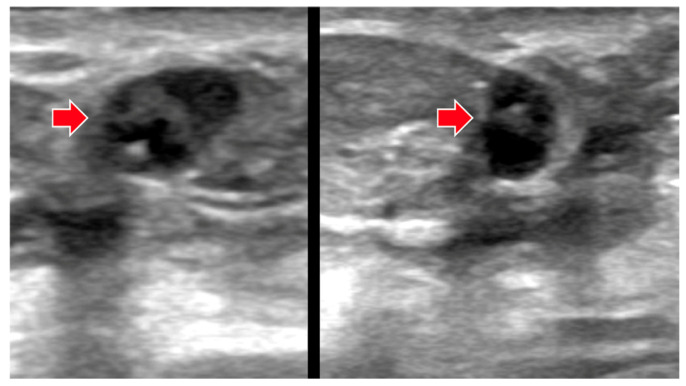
**False negative BI-RADS and AI assessment of mass due to DCIS**. Transverse (**left**) and sagittal (**right**) US images directed to a new mammographic mass in this 77-year-old woman show a mixed cystic and solid nonparallel 6-mm mass (arrows) that had clinically been assessed as BI-RADS 3. In the training set, the KDS score was 0.39 (a probably benign rating). US-guided core biopsy six months later showed intermediate nuclear grade DCIS, ER/PR negative.

Adding the KDS score significantly (*p* < 0.001) improved BI-RADS-based cancer prediction with or without adjusting for age (or other factors from Table 1, which were not significant after accounting for BI-RADS). The resulting combined assessment (KDS + BI-RADS) had an ROC curve with an AUC of 0.98 (CI: 0.97–0.99) and achieved specificity of 75.2% (785/1044) at the targeted sensitivity of 98.0% (394/402), Figure 4, Table 2. The 98–sensitivity threshold estimated for combined KDS + BI-RADS (fixed for the later validation assessment) implied decisions that adjusted BI-RADS-based recalls in non-extreme categories, i.e., BI-RADS 3 to 4C (Table 3, with only categories 3 and 4A actually affected). In the training subset there were 5% (21/402) cancer cases where binary classifications of KDS alone and BI-RADS alone disagreed. One DCIS was misclassified by both (Figure 5).

### 3.3. Validation Performance

In the validation set of 325 cases, including 95 (29.2%) malignancies, standalone KDS had an AUC of 0.97 (CI: 0.95–0.98) and achieved sensitivity of 98% (93/95, CI: 95–100%) and specificity of 70.9% (163/230, CI: 65.0–76.7%) at the default threshold of 0.5. At the training estimated KDS threshold of 0.23 for 98% sensitivity, KDS achieved sensitivity of 100% (95/95, CI: 96–100%) and specificity of 50.8% (117/230, CI: 44–57%), thus being more conservative than the default threshold of 0.5. BI-RADS assessments had a similar AUC of 0.95 (CI: 0.93–0.97) and somewhat lower specificity of 63.9% (147/230, *p* = 0.10) with the same sensitivity of 98% (93/95) for clinically actionable diagnostic findings (i.e., BI-RADS 4A or higher). The derived combined KDS + BI-RADS assessment, compared to BI-RADS alone, had a significantly higher AUC of 0.98 (95% CI: 0.97–1.00, *p* < 0.001) and achieved significantly higher specificity of 74.4% (171/230, CI: 68.7–80.0%, *p* < 0.001), or, equivalently, a more than 25% reduction in false positive rates (from 0.36 to 0.26), with the same sensitivity of 98% (93/95, CI: 95.0–100%) at the pre-defined threshold.

The relatively superior characteristics of the combined KDS + BI-RADS compared to BI-RADS alone prevailed in the validation subsets of small (≤10 mm) and large (>10 mm) lesions, women under and over 50 years old, and women with or without symptoms (Table 3).

In classification of lesions with specific BI-RADS from the validation set (Table 4), KDS at a default threshold of 0.5 was able to identify two cancers assessed BI-RADS 2 or 3, respectively, but missed two grade 3 TNBC cancers assessed as BI-RADS 4B (Figure 2 and Figure 3), while maintaining the same cancer detections for other BI-RADS assessments (i.e., discordance was 4/95 of malignancies, 4%). KDS prompted multiple unnecessary recalls of benign masses with initial BI-RADS 2 and 3 (21/106 and 19/41, respectively), which were overcompensated by a substantial reduction of false-positive recalls for BI-RADS 4A and 4B (53/77 and 3/6, respectively). But the net reduction in false-positive recalls was short of statistical significance (0.29, 67/230, with KDS alone versus 0.36, 83/230, for BI-RADS alone *p* = 0.10).

In contrast, as shown in Table 4, derived combined KDS + BI-RADS assessments replicated the clinical BI-RADS decisions for all cancers in the validation set (by design for BI-RADS 2 and 5, and for other BI-RADS assessments due to the high KDS-estimated probability of malignancy for cancers in our sample). At the same time, combined KDS + BI-RADS substantially reduced unnecessary recalls for benign masses assessed as BI-RADS 4A (for those cases when the KDS-estimated probability was lower than 14%), confirmed the BI-RADS recalls for six non-cancers assessed as BI-RADS 4B, and unnecessarily recalled two of 41 non-cancers assessed as BI-RADS 3 (because of their KDS estimated probability of malignancy being greater than 69%). Despite the imperfections, this proof-of-concept combined assessment (KDS + BI-RADS) achieved a significantly lower net recall rate than the clinical BI-RADS recommendations alone (0.26, 59/230, versus 0.36, 83/230, *p* < 0.001) without sacrificing cancer detections.

### 3.4. Impact of KDS on Benign, Probably Benign, and Low-Suspicion Masses

Table 4 provides details of lesions given BI-RADS assessments of 2, 3, or 4A, in the validation set, including 18 malignant and 224 benign masses. The sensitivity of BI-RADS in this subset was 89% (16/18), and of standalone KDS, 100% (18/18). The specificity of BI-RADS was 66% (147/224) versus 71% (160/224), *p* = 0.18, for standalone KDS.

## 4. Discussion

Pre-trained KDS added to BI-RADS significantly outperformed breast imaging specialists’ BI-RADS assessments alone, resulting in substantial improvement in accurate identification of benign cases (i.e., a more than 25% reduction in the false positive rate at sensitivity of 98% in the validation dataset). In validation, standalone KDS performance was comparable to BI-RADS, with an AUC of 0.97 (vs. 0.95 for BI-RADS alone), with somewhat higher (but not statistically significantly different) specificity (71% vs. 64% for BI-RADS) and the same sensitivity (98%) at the default threshold of 0.5.

In a nonoverlapping set of 319 lesions from screening US, including 88 (27.6%) malignancies, a prior version of KDS software (v. 1.3) showed standalone AUC of only 0.77 (95%CI 0.72–0.83) [25]; nine specialist breast imaging radiologists did not show improvement from a baseline mean AUC of 0.82 when adding KDS. When KDS scores were artificially improved, particularly with improved specificity, then radiologists did benefit from KDS. In that study, there were seven circumscribed malignancies, and KDS classified 4/7 (57%) of those as suspicious. Since that version, KDS has undergone continued training and now also can provide standard lesion description, though it does not include the special cases of cyst, complicated cysts with debris, clustered microcysts, or intraductal masses, and it has not been trained on non-mass lesions or lymph nodes.

Coffey et al. [26] evaluated KDS (v. 2.1) for 345 TNBC and showed moderate agreement with radiologists on lesion shape and orientation. Radiologists recommended biopsy for 339/345 (98.3%) TNBC; 333/345 (96.5%) were deemed suspicious by KDS, including the six lesions that were false negative for the radiologists. In our series, KDS at a default threshold of 0.5 was able to identify two cancers assessed as BI-RADS 2 or 3, respectively, but missed two cancers assessed as BI-RADS 4B.

The performance of clinical BI-RADS was very high (AUC > 0.95) in this series but left room for improvement in accuracy in both training (with 61% specificity at sensitivity of 98%) and validation subsets (with 64% specificity at sensitivity of 98%). BI-RADS assessments missed fewer benign-appearing cancers than KDS, likely because there was additional information available: the US may have been directed to an enhancing mass on MRI, or the mass was known to be a new finding mammographically or clinically. Overall, 54/475 (11.4%) TNBC 2 cm or smaller in our series had relatively benign features on US, and eight [of 17 (47%)] of KDS misclassifications of TNBC as benign or probably benign were among those 54 TNBC with benign features. Kuzmiak et al. [27] found 44/67 (66%) premenopausal TNBC had mammographically round/oval shape, significantly more than those of postmenopausal women at 64/129 (49.6%, *p* = 0.041). Only 8/67 (11%) of premenopausal TNBC had circumscribed margins (as did only 3/196, 2.3% of those in postmenopausal women), and only 6/197 (3.0%) of all sonographically visible TNBC had circumscribed margins in their series. Benign features were more common in Black than White women, but differences were not significant [27]. In a recent, nonoverlapping review, 109/559 (19%) of TNBC were circumscribed on US [22].

In the training subset, there were 5% (21/402) cancer cases where binary classifications of KDS alone and BI-RADS alone disagreed, and in the validation subset, 4% (4/95) were discordant; one DCIS was missed by both. At the same time, the defined proof-of-concept KDS + BIRADS combination completely agreed with the clinical BI-RADS assessments of cancers, while substantially improving the specificity (by inducing a BI-RADS-dependent threshold for KDS). Thus, optimal combinations of this type could be used to improve accuracy of clinical management. Furthermore, radiologists using KDS could utilize the available information, including consideration of their initial BI-RADS assessment (demonstrated by the proof-of-concept combination), to achieve even more substantial improvements in performance.

Reduced false positives with standalone KDS in this study may be otherwise difficult to realize in clinical practice. While standalone KDS accuracy overall was not different from specialist radiologists, even in the validation set, KDS missed a few cancers originally assessed as BI-RADS 4B. Most of the potential benefit was downgrading benign BI-RADS 4A, which is unlikely to be accepted by breast radiologists in the absence of 100% sensitivity. For example, in previous work, breast radiologists were more inclined to alter assessments based on KDS scores in artificially high-sensitivity or high-specificity modes [25]. In contrast to standalone KDS, the combined KDS + BI-RADS assessment, at a threshold of 98% sensitivity, replicated the BI-RADS assessments for cancers, while still providing substantial reduction in BI-RADS 4A false positives. Making use of KDS in addition to (rather than instead of) clinical BI-RADS assessment appears promising for clinical practice, and, as for any method used to improve specificity, one would expect only to make adjustments to management of lesions near the threshold for biopsy, i.e., BI-RADS 3 or 4A masses.

AI is more likely to benefit practitioners with less subspecialty expertise. Mango et al. [23] found that KDS improved interpretive performance most among physicians without fellowship training in breast imaging. US is very effective in evaluating breast symptoms [28,29]. Triage of palpable lumps in low-resource settings [30] is a promising use of US AI that is likely to be increasingly effective with improvements in low-cost portable US image quality. We found KDS performance to be slightly better in symptomatic cases in this series.

There are several possible methods to reduce false positives from screening US, including elastography and AI. Cysts and noncalcified fibroadenomas are typically soft on elastography without effects on the surrounding tissue [31]; invasive carcinomas typically show a surrounding rim of stiffness [32] and may also show internal stiffness. In several prospective multicenter studies, use of elastography has been proven to have the potential to reduce biopsy of benign BI-RADS 4A masses and to help identify the few malignancies otherwise assessed as BI-RADS 3 without loss of sensitivity [33,34]. Despite widespread availability on current US systems, routine breast elastography has not become standard practice in the United States.

One of the strengths of our work is the use of images from multiple manufacturers and with input from multiple breast imaging specialists. We emphasized lesions near the threshold of clinical decision making. There are several limitations to our work, however. We did not evaluate lesion detection, as KDS software is not used for lesion detection. Radiologists reviewing lesion features were not blinded to the outcomes. The BI-RADS assessments used were from a mix of clinical assessments and highly experienced breast-imaging radiologists; clinical BI-RADS assessments were often influenced by the method of detection, such as screening MRI. We imputed results from the combination of BI-RADS assessments and KDS—there was no radiologist re-reading with the KDS input. Continued clinical validation of outcomes is needed and is ongoing, as updated commercially available KDS incorporating these cases received FDA approval on 15 November 2024 (v. 3.6) and is deployed at over 70 clinical sites internationally. Finally, our study was of 2D handheld US images in orthogonal views and does not generalize to automated ultrasound, though KDS has been validated for such.

In conclusion, overall performance of AI (KDS) on sonographic breast masses was very similar to the performance of clinical BI-RADS assessments in our study. However, in this highly selected case set of masses 2 cm or smaller, enriched in TNBC, benign, and probably benign masses, standalone KDS missed cancers initially detected by BI-RADS, including two assessed as BI-RADS 4B in the validation set. Using KDS in addition to BI-RADS assessments (via derived KDS + BI-RADS at 98% sensitivity) allowed us to replicate the BI-RADS decisions for cancers while improving specificity (effectively tailoring the threshold for KDS based on BI-RADS assessment). Further study that modifies the AI output based on the radiologist’s initial BI-RADS assessments may help integrate US AI into practice, and is ongoing.

## Figures and Tables

**Table 1:** Demographic and lesion characteristics for 1771 breast masses in 1771 women.

Characteristic	Of Total 1771	Benign (1274)		Malignant (497)	
		#	%	#	%
Age (*p* < 0.001)					
<40	187 *	138	11%	49	9.9%
40–49	514	448	35%	66	13%
50–59	506	372	29%	134	27%
60–69	353	217	17%	136	27%
70–79	163	82	6.4%	81	16%
≥80	48	17	1.3%	31	6.2%
Race (*p* < 0.001)					
Asian	75	69	5.4%	6	1.2%
Black	188	97	7.6%	91	18%
Other	362	261	20%	101	20%
White	1146	847	66%	299	60%
Lesion size (*p* < 0.001)					
≤10 mm	1185	975	77%	210	42%
>10 mm	586	299	23%	287	58%
Symptomatic (*p* < 0.001)					
No	1338	1044	82%	294	59%
Yes	433	230	18%	203	41%
BI-RADS Assessment (*p* < 0.001)					
2	565	563	44%	2	0.4%
3	222	218	17%	4	0.8%
4A	527	453	36%	74	15%
4B	175	30	2.4%	145	29%
4C	119	10	0.8%	109	22%
5	163	0	0%	163	33%

* includes 35 missing (5 in validation set). Percentages are column percentages.

**Table 2:** Overall and classification performance of BI-RADS and KDS AI in the training and validation subsets of 1771 breast masses.

Dataset (Benign + Malignant)		AUC		Sensitivity * (%)		Specificity * (%)	
	Marker	Est.	95%CI	Est.	95%CI	Est.	95%CI
Training	BI-RADS	0.96	(0.95, 0.97)	99.0	(98.0, 100.0)	60.7	(57.8, 63.7)
(1044 + 402)	KDS	0.96	(0.95, 0.98)	95.3	(92.9, 97.2)	77.8	(75.3, 80.3)
	KDS + BIRADS	0.98	(0.97, 0.99)	98.0	(97.0, 99.5)	75.2	(71.6, 76.9)
Validation	BI-RADS	0.95	(0.93, 0.97)	98.0	(95.0, 100)	63.9	(57.7, 70.1)
(230 + 95)	KDS	0.97	(0.95, 0.98)	98.0	(95.0, 100)	70.9	(65.0, 76.7)
	KDS + BIRADS	0.98	(0.97, 1.00)	98.0	(95.0, 100)	74.4	(68.7, 80.0)

* Default positivity thresholds of 4A or higher for BI-RADS and ≥0.5 for KDS alone were used, with the threshold for the combined KDS + BIRADS assessment estimated to achieve sensitivity of 98% in the training set (and kept fixed for the validation set). Est. = estimate.

**Table 3:** Overall performance within clinically relevant subgroups of the validation set of 325 masses.

Factor	Benign + Malignant	BI-RADS		KDS		KDS + BIRADS *	
		AUC	95%CI	AUC	95%CI	AUC	95%CI
Lesion size							
≤10 mm	(168 + 41)	0.93	(0.88, 0.97)	0.95	(0.91, 0.98)	0.96	(0.93, 1.00)
>10 mm	(62 + 54)	0.97	(0.94, 0.99)	0.98	(0.96, 1.00)	1.00	(0.99, 1.00)
Age							
<50 years	(101 + 21)	0.92	(0.87, 0.97)	0.96	(0.92, 1.00)	0.98	(0.97, 1.00)
≥50 years	(124 + 74)	0.96	(0.94, 0.99)	0.97	(0.95, 0.99)	0.98	(0.96, 1.00)
Symptoms							
No	(187 + 62)	0.95	(0.92, 0.98)	0.96	(0.94, 0.98)	0.97	(0.95, 1.00)
Yes	(43 + 33)	0.96	(0.93, 0.99)	0.98	(0.96, 1.00)	1.00	NA

* Based on the combination estimated in the training set. NA = not applicable.

**Table 4:** Classification performance of BI-RADS and KDS-based decisions for 325 masses in the validation set.

			BI-RADS		KDS Alone		KDS + BI-RADS		
			Positivity at ≥4A		Positivity at ≥0.5		Positivity at the Triaging Threshold *		
Lesions	BI-RADS	Total	N	%	N	%	N	%	Implied Positivity Threshold for KDS
Benign Masses	Overall	230	83	36%	67	29%	59	26%	
	2	106	0	0%	21	20%	0	0%	>1.00
	3	41	0	0%	19	46%	2	5%	>0.69
	4A	77	77	100%	24	31%	51	66%	>0.14
	4B	6	6	100%	3	50%	6	100%	>0.09
Malignant Masses	Overall	95	93	98%	93	98%	93	98%	
	2	1	0	0%	1	100%	0	0%	>1.00
	3	1	0	0%	1	100%	0	0%	>0.69
	4A	16	16	100%	16	100%	16	100%	>0.14
	4B	29	29	100%	27	93%	29	100%	>0.09
	4C	22	22	100%	22	100%	22	100%	>0.04
	5	26	26	100%	26	100%	26	100%	>0.00

* Estimated for achieving 98% sensitivity in the training set (kept fixed in the validation set).

## Data Availability

Because the images were used in proprietary software development, we are not able to provide them. The Koios DS software used in this study is commercially available.
